# The Relationship of Sphingosine Kinase 1 With Pyroptosis Provides a New Strategy for Tumor Therapy

**DOI:** 10.3389/fimmu.2020.574990

**Published:** 2020-10-02

**Authors:** Xianwang Wang, Yue Yang, Wen-Qi Cai, Yingying Lu

**Affiliations:** ^1^Department of Biochemistry and Molecular Biology, Health Science Center, Yangtze University, Jingzhou, China; ^2^The Seventh Affiliated Hospital, Sun Yat-sen University, Shenzhen, China

**Keywords:** spingosine kinase 1, pyroptosis, calcium and integrin-binding protein 1, cancer, sphingosine-1-phosphate

## Abstract

Sphingosine kinase 1 (SPHK1) is a crucial molecule that catalyzes sphingosine to synthesize sphingosine-1-phosphate (S1P), facilitating cell survival signaling. Pyroptosis is a perplexing inflammatory mode of cell death primarily triggered by caspase-1, evoked by the NLRP3 inflammasome. Sphingosine is identified as a danger-associated molecular pattern (DAMP), which activates the NLRP3 inflammasome assembly and induces the pyroptosis. It has been demonstrated that macrophages play a pro-tumorigenic role and are closely associated with tumor progression. Attenuation of SPHK1 activity contributes significantly to macrophage pyroptosis and tumor inhibition. Calcium and integrin-binding protein 1 (CIB1) plays an important role in the translocation of SPHK1 from the cytoplasm to the plasma membrane, whereas CIB2 blocks the subcellular trafficking of SPHK1. Therefore, knockout of CIB1 or over-expression of CIB2 will result in sphingosine accumulation and contribute significantly to cancer treatment by several approaches. First, it directly provokes cancer cell apoptosis or triggers robust anti-tumor immunity by pyroptosis-induced inflammation. Second, it could restrain SPHK1 translocation from the cytoplasm to the plasma membrane and further pyroptosis, which not only drive M2 macrophages death but also facilitate tumor microenvironment inflammation as well as the further release of sphingosine from damaged macrophages. The perspective might provide novel insight into the association between SPHK1 and pyroptosis and suggest the potential target for cancer therapy.

## Introduction

Sphingolipids play increasingly important roles in the regulation of cell fate determination. Sphingosine kinase 1 (SPHK1) is a pivotal kinase that catalyzes ceramide and sphingosine (Sph) to yield a key sphingolipid signaling mediator sphingosine-1-phosphate (S1P), which stimulates multiple physiological processes, including cell growth, proliferation, survival, inflammation, migration, angiogenesis, etc. (1–4). Sphingosine kinase 2 (SPHK2), an isozyme of SPHK1, also has similar biological activity (**Table 1**). Conversely, ceramide, a second messenger in sphingolipid metabolism, triggers anti-proliferative responses involving growth inhibition, apoptosis, differentiation, and senescence (17, 18). Sph, generated from ceramide, also involves cell growth arrest and apoptosis (19).

**Table 1:** The comparison between SPHK1 and SPHK2.

Name	SPHK1	SPHK2	References
Location	Cell membrane Endosome membrane Nucleus	Nucleus, Cytoplasm	(5–15)
Transport	CIB1/CIB2		(6, 7)
Metabolism	Cleavage of sphingomyelin by sphingomyelinase generates ceramide that can promote apoptosis, cell-cycle arrest, and cellular senescence, and then ceramide can be cleaved by ceramidase to produce sphingosine that can be phosphorylated by SPHK1/2 to form S1P.		(4)
Potential Mechanism	Decreases intracellular ceramide levels, enhances cell growth, and inhibits apoptosis.	Increases intracellular ceramide levels, inhibits cell growth, and enhances apoptosis.	(16)

**Figure 1:**
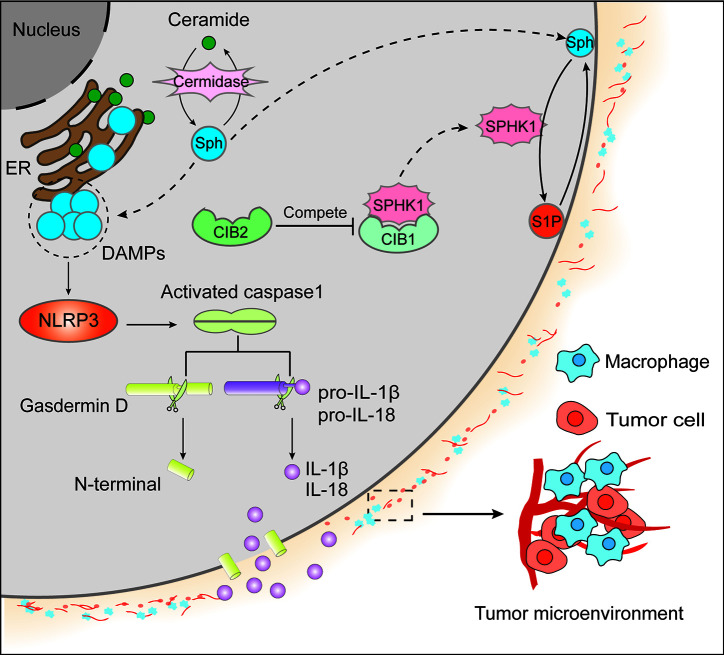
Ceramide, primarily located in the endoplasmic reticulum membrane, generates sphingosine (Sph) by ceramidase. Both ceramide and Sph could contribute to cell apoptosis. On the one hand, Sph migrates to the cytomembrane from the endoplasmic reticulum (ER) membrane and produces sphingosine-1-phosphate (S1P) by sphingosine kinase 1 (SPHK1). In contrast to the apoptotic effect of Sph, S1P could facilitate cell survival signaling. On the other hand, SPHK1 subcellular localization was regulated by calcium and integrin-binding protein 1 (CIB1) and CIB2. It is believed that the translocation of SPHK1 from the cytoplasm to the cytomembrane is dependent on CIB1, whereas CIB2 competes with CIB1 to binds to SPHK1, resulting in the cytoplasm retention of SPHK1 and the inhibition of its enzymatic activity. Additionally, the accumulation of Sph in ER acts as DAMPs to activate NLRP3 and the oligomeric RLRP3 inflammasome, further causing caspase-1 homo activation and activated caspase-1 cleave gasdermin D to form N-terminal and pro-IL-1β/pro-IL-18 to yield IL-1β/IL-18, respectively. Then N-terminal of gasdermin D will form the channels in the plasma membrane causing massive leakage of cytosolic contents including IL-1β and IL-18, which leads to tumor microenvironment inflammation in that IL-1β can activate primary T cells and memory T cells, IL-18 can promote interferon (IFN)-γ production in TH1 cells, NK cells and cytotoxic T cells, boost the development of TH2 cells, and improve local inflammation response.

Zychlinsky and colleagues observed a lytic form of cell death in *Shigella flexneri*–infected macrophages referred to pyroptosis (20, 21). Cumulating evidence has acknowledged that pyroptosis is very perplexing, appears to be multifactorial, and is a pattern of inflammatory programmed cell death pathway activated by caspase-1, caspase-4, and caspase-5, or caspase-11. It is a pathway of cell death characterized by pore formation in the plasma membrane, cell swelling and rupture of the membrane, and massive leakage of cytosolic contents (22, 23). Caspase-1, a multifunctional inflammatory mediator, manages host defense against bacteria, tissue repair, tumorigenesis, metabolism, and membrane biogenesis (24). Caspase-1 is evoked by NLRP3 (Nod-like Receptor Protein 3) inflammasome, which is a multiprotein complex consisting of NLRP3 (a cytosolic mediator molecule), adaptor protein ASC (an apoptosis-associated speck-like protein containing caspase recruitment activation domain), and an effector molecule cysteine protease pro-caspase-1. These perplexing particles would be generated on exposure to cellular perturbations of intracellular microbial infections (21, 25–27). Moreover, in macrophages, priming and activation are the two essential steps for NLRP3 inflammasome activation. The priming stage is elicited by inflammatory stimuli such as TLR4 agonists, which initiates NF-κB-induced NLRP3 and pro-IL-1β expression by resulting in transcriptional regulation of many inflammatory genes, involving cytokines and inflammasome components, and the activation step is driven by DAMPs (danger-associated molecular patterns) and PAMPs (pathogen-related molecular patterns), thereby facilitating NLRP3 inflammasome formation and caspase-1-induced pyroptosis (28–31). On activation of NLRP3 inflammasome, caspase-1 mediates macrophages pyroptosis, which is controlled by the N-terminal domain of gasdermin D (GSDMD) *via* assembling channels in the cell membrane and activating pro-IL-1β and pro-IL-18 for their secretion from the cells (**Figure 1**) (32).

A new mechanism of macrophage pyroptosis can be triggered by NETs (neutrophil extracellular trap) released by HMGB1 (high-mobility group box1) in sepsis, which can release nuclear contents of the PMN (polymorphonuclear neutrophils) into the extracellular space to trap and kill the bacteria in responding to pathogens infection (33). However, lipopolysaccharide or Gram-negative bacteria can trigger pyroptosis known as NETosis that in neutrophils, activating GSDMD cause neutrophils pyroptosis through caspase-4/11 noncanonical inflammasome signal pathway (34). Furthermore, pyroptosis has an important influence on the progress of many different neoplasms. In particular, tri-negative breast cancer, Barrett’s esophageal cancer, and A549 cells can induce the occurrence of pyroptosis *via* caspase-1 canonical inflammasome signal pathway stimulators (35, 36). Especially in HCC, the expression level of NLRP3, caspase-1, IL-1β, and IL-18 was significantly attenuated compared with that in adjacent normal tissues. Noticeably, there was a negative correlation between the pathological progress of HCC and the expression level of NLRP3 inflammasome (37–39).

Pyroptosis is a complex cell death mechanism involving multiple stimulators, pathway in which cell cytokines and inflammatory cytokines take part. Therefore, considering pyroptosis facilitation of tumor treatment requires more profound exploration. There is a balance between pro- and anti-tumorigenic roles of pyroptosis. On the one hand, pyroptosis can yield a certain microenvironment for tumorigenesis, tumor growth, and progression. On the other hand, the induction of TAMs pyroptosis can arrest tumor cell growth and development. Pyroptosis of cancer cells is also believed a potential tumor treatment approach. Thus, pro- and anti-tumorigenic roles of pyroptosis need further exploration owning to the double-edged sword of pyroptosis. In this study, the relationship of sphingosine kinase 1 on pyroptosis as it provides a new strategy for tumor therapy was discussed.

## Sph Acting as DAMP Contributes to the Pyroptosis

Luheshi et al. identified that both sphingosine and sphingosine analog, FTY720 can induce NLRP3-dependent activation of caspase-1 and secretion of IL-1β from LPS-primed peritoneal macrophages *in vitro*, which was contributed to lysosomal membrane rupture, demonstrated by the translocation of cathepsin B from lysosomes to the cytosol (40). Moreover, it is revealed that cathepsin B is diffuse into the cytosol when lysosome is disrupted, accelerating a conversion in the specificity of the enzyme from an exopeptidase to an endopeptidase, efficiently cleaving SPHK1 at various sites, and leading to loss of the protein and subsequent frustration of SPHK1 activity (41). Therefore, Sph inducing lysosomal membrane rupture and resulting in the translocation of cathepsin B to the cytosol from lysosomes confers two effects. On the one hand, cathepsin B in cytoplasm cleaves SPHK1 impairing the conversion into S1P and accumulation of Sph; on the other hand, cumulative Sph acts as a DAMP, activating NLRP3 inflammasome assembly, thereby inducing pyroptosis. There are two common ways for macrophages to be activated: IL-4 mediated alternative macrophage activation, which is known as alternatively activated (M2) macrophages, and classically activated (M1) macrophages activated by microbe products or PAMPs (42–44). Recently researchers have characterized that tumor-associated macrophages (TAMs, M2-like functions, and phenotype) permeate into tumor tissues in large numbers, and macrophages have a pro-tumorigenic role closely associated with tumor promotion (45). Thus, inhibiting the enzymatic activity of SPHK1 might contribute greatly to TAMs pyroptosis to promote anti-tumor progression.

## CIB1 Modulates the Translocation of SPHK1

Calcium and integrin-binding protein 1 (CIB1), first discovered in 1997 as a novel interacting protein of the integrin aIIb, is a calcium modulating molecule, which is implicated in multiple cellular processes, including calcium signaling, cell growth, and proliferation, migration, adhesion, and apoptosis (46, 47). The myristoyl group of CIB1 is sequestered into a hydrophobic pocket in the protein’s absence of intracellular Ca^2+^. Ca^2+^ binding promotes topology changes enabling two effects as follows: first in conferring the association between protein and partners, and second to provoke the appearance of the myristoyl group from its original buried region, targeting the protein and any nascent associated interacting partner to intracellular membranes. Notably, CIB1 is essential for the agonist-induced migration of SPHK1 from the cytoplasm to the plasma membrane (5, 48). Jarman et al. explored how either knockdown of CIB1 or a dominant-negative CIB1 could restrain the agonist-dependent redistribution of SPHK1 (5). Zhu et al. also confirmed that over-expression of CIB1 contributes high distribution of SPHK1 to the plasma membrane, which was verified by boosted plasma membrane-associated SPHK1 activity, and attenuates the synthesis of S1P in CIB1 over-expressing cells (6). Although both CIB1 and CIB2 bind to SPHK1 on the α8 helix of SPHK1, CIB2 lacks the Ca^2+^-myristoyl switch function. Contrary to CIB1, CIB2 blocks the translocation of SPHK1 to the cell membrane and undermines its subsequent signaling. The interaction between CIB2 and SPHK1 is independent of Ca^2+^, or Mg^2+^, and myristoylation of CIB2 does not affect their association (6, 7). It is probable that CIB2 competitively binds to SPHK1 with CIB1 and gives rise to the retention of SPHK1 in the cytoplasm.

## Summary and Prospect

Thus, a proposal that facilitated Sph release in tumor microenvironment might be an effective treatment scheme. Baroja-Mazo et al. found that NLRP3 and ASC complexes released from pyroptosis cells functioned as danger signals giving rise to amplifying inflammation by improving the activation of extracellular caspase-1 and in surrounding macrophages following internalization of the particles (16).

In summary, mutating CIB1 or over-expression of CIB2 resulting in Sph accumulation might contribute twofold to cancer therapy: in cancer cells, it directly provokes cell apoptosis, or triggers robust anti-tumor immunity by pyroptosis-induced inflammation as well as a combination with some potential anti-tumor drugs; in M2 macrophages, it could restrict SPHK1 redistribution from cytoplasm to cytomembrane and trigger subsequent pyroptosis, which not only causes M2 macrophages death and play an essential role in anti-tumor progression, but also facilitates both tumor microenvironment inflammation and Sph leakage from damaged macrophages. Together, our review might provide insight into the close association between SPHK1 and pyroptosis and contribute to tumor treatment’s potential strategy.

## Author Contributions

XW and YL designed the study. XW and YY drafted the manuscript. YY and W-QC drew the figure and filled the table. XW and YL and YY revised the manuscript. All authors contributed to the article and approved the submitted version.

## Funding

This work was supported by grants from the National Natural Science Foundation of China (31700736, 31501116), China Scholarship Council (201908420102), Hubei Medical Youth Tip-Top Talent (to XW), Leading Talent Program of Yangtze Talent Project (to XW), and the College Students Innovative Entrepreneurial Training Program in Yangtze University (2018184, 2019372).

## Conflict of Interest

The authors declare that the research was conducted in the absence of any commercial or financial relationships that could be construed as a potential conflict of interest.
